# Relationships Between the Life Satisfaction, Meaning in Life, Hope and COVID-19 Fear for Turkish Adults During the COVID-19 Outbreak

**DOI:** 10.3389/fpsyg.2021.633384

**Published:** 2021-03-11

**Authors:** Zeynep Karataş, Kıvanç Uzun, Özlem Tagay

**Affiliations:** Burdur Mehmet Akif Ersoy University, Burdur, Turkey

**Keywords:** life satisfaction, meaning in life, hope, COVID-19 fear, adults

## Abstract

The current study investigated whether there are significant relationships between life satisfaction and meaning in life, hope and COVID-19 fear and the extent to which life satisfaction is predicted by these variables. The study group of this research consists of 1,186 adults with the mean age of 41.04. Study group participants are consists of different cities of different regions of Turkey. As the data collection tools, the life satisfaction scale, the meaning in life scale, the dispositional hope scale and the COVID-19 fear scale were used. The results of the analyses have revealed that meaning in life, hope (actuating thinking and alternative ways thinking) are significant predictors of life satisfaction as positively. Besides, it is seen COVID-19 fear, age, gender and the presence of people infected with COVID-19 around aren’t significant predictors of life satisfaction in adults.

## Introduction

As a result of cases of unknown etiology seen in Wuhan City, of China in December 2019, Severe Acute Respiratory Syndrome; that is, Corona disease was named as COVID-19 by the World Health Organization (WHO) (Wang C. et al., 2020). Many countries in the world have set restrictions and taken policy measures within the context of social withdrawal, distance working and self-isolation. Severe economic repercussions have occurred. Because of the COVID-19 outbreak, people’s daily work activities have deteriorated, and reasons such as reduced social interactions and difficulty in adapting to the new situation have also brought about psychological problems for many individuals. Individuals who cannot access social support networks when they need it cannot meet their congenital attachment and acceptance needs. This may lead to psychological and emotional disorders such as depression, anxiety, substance abuse and suicidal ideation throughout the society (Godinic et al., 2020). According to the data published on the website of the WHO, it is seen that there were 54.301.156 confirmed cases and 1.316.994 deaths worldwide within the scope of the corona virus outbreak on November 16, 2020^1^ (World Health Organization [WHO], 2020a). On the same date by the health ministry confirmed cases in Turkey, the total number of 417.594, while the number of deaths has been reported as a total 11.601. The first officially reported COVID-19 case in Turkey was on March 10, 2020 and the numbers have escalated rapidly. The Turkish government urged citizens to stay at home except for necessities and urgencies. Currently, Turkey has the most confirmed COVID-19 cases outside of Europe and the United States, leaving China and Iran behind (World Health Organization [WHO], 2020b).

In coronavirus pandemic people’s feelings and thoughts about the social world and their own lives can be negatively affected by the process. People can be worried about losing their jobs and going out and have to change their old social life habits, and may be afraid of being infected by any person they encounter. Fear refers to a normal reaction to an emerging threat that prepares the individual to react acutely to possible physical and mental harm (Pappas et al., 2009). Fear is an adaptive emotion that serves to mobilize energy to deal with potential threat. However, when fear is not well calibrated to the actual threat, it can be maladaptive. For instance, when fear is too excessive, this may have detrimental effects both at the individual and societal level. On the other hand, when there is insufficient fear, this may also result in harm for individuals and society (Deacon and Maack, 2008; Olatunji et al., 2011; in cited Mertens et al., 2020).

The coronavirus pandemic makes people feel scared, uncertain and anxious (Jerzy et al., 2020). In the event of fear, the person can concentrate on the death news caused by COVID-19 and can infect his/her family members and environment with this fear. Thoughts of getting sick during an epidemic also increase fear. The intensity of negative thoughts that arise during a pandemic also causes negative emotions such as fear. On the other hand, fear is also accepted as an emotion that has functional and evolutionary importance and can be encountered anywhere (Ho et al., 2020; Presti et al., 2020).

The good life necessarily entails well-being. The psychology of well-being serves as an umbrella term for happiness, health, flourishing, and optimal functioning at both the individual and national levels in both positive and negative conditions. Given that there are cultural differences, well-being still provides a useful index on how we are doing and how well we live at the individual and national level (Wong, 2011). Diener and Tov (2009) have reported that overall life satisfaction and positive effects have different predictors in different countries. Well-being reflects not only healthy functioning and happiness (Ryan and Huta, 2009), but also serves an evaluative function in the self-determination process (Ryan et al., 2008). Pandemic effects well-being; it is important to look for correlates which could serve as protective or risk factors; fear was chosen as a possible risk factor, hope and meaning in life, as possible protective factors.

During the pandemic period, the effect of positive psychology concepts on well-being has become even more important. Positive Psychology 2.0 (PP 2.0), as conceptualized by Wong (2011), proposed that the most promising strategy to accomplish the mission of positive psychology is to confront the dark side of human existence and understand the unique experience and expression of well-being in different cultures. Thus, PP 2.0 emphasized the existential universal on one hand, and indigenous cultural expression on the other hand.

The recent contributions of positive psychology present the quality of life as a fundamental indicator for health promotion and prevention strategies (Santisi et al., 2020). According to PP 2.0 (Wong, 2011), well-being is not the algebra of positive minus negative but positive plus negative. In other words, the capacity to transcend and transform negative provides an additional source of well-being to positively based well-being. Well-being enriches and energizes life; it also endows life with a sense of joy and meaning. All the struggles and sufferings seem worth it when we are able to drink from the fountains of happiness. It is tempting to view the meaning in life purely in terms of positive effects. However, psychology of well-being needs to study both the perils of happiness (Wong, 2007) and the benefits of suffering (Frankl, 1963; Wong, 2009).

Subjective well-being is one of the important concepts of positive psychology. It is the personal perception and experience of positive and negative emotional responses and global and (domain) specific cognitive evaluations of satisfaction with life. Life satisfaction has been defined as “a person’s cognitive and affective evaluations of his or her life” (Diener et al., 2002). Life satisfaction, meaning in life and hope are some of the most important factors that affect the individual’s thoughts and feelings in situations of danger. In addition, these factors affect how the current situation and the future are evaluated if the danger continues for a long time and people’s order of life starts to deteriorate. Higher meaning in life and hope associated by a high level of life satisfaction can help people cope with dangerous irregularities (Abrams et al., 2005; Batthyany and Russo-Netzer, 2014). In the literature, there are studies investigating the relationship of life satisfaction with life quality (Manning-Walsh, 2005); with hope and personality characteristics (Halama, 2010); with happiness, depression, hope and meaning in life (Nasiri and Bahram, 2008); with humor and sense of gratitude (Proyer et al., 2013); with perfectionism and humor (Çalışandemir and Tagay, 2015); with social support, self-esteem and gender roles (Matud et al., 2014); with sensitivity to increasing social risk and negative feelings (Li et al., 2020).

Hope is considered an important determinant of positive development. Hope is defined as the person’s belief in his/her capacity to achieve his/her goals, pondering about his/her goals and proceeding toward them (Snyder, 2002). In the literature, hope was found to be correlated with life satisfaction (Valle et al., 2004) and personal adaptation and psychological well-being (Gilman and Huebner, 2006). It was also related to optimism and life satisfaction (Ciarrochi et al., 2007); meaning in life (Feldman and Snyder, 2005) and resilience (Wu, 2011; Duggal et al., 2016).

People seek ways of understanding themselves and the world and exhibit cognitive and behavioral activities to accomplish this (Higgins, 2000). When people theoretically understand themselves and the world, why they are in the world and determine what they want in their lives, they experience the existence of meaning. In this way, people can find meaning in life. Meaning in life is defined as the strength and intensity of the efforts made by people to understand and/or increase the meaning, importance and purpose in their lives (Steger et al., 2008). PP 2.0 adds depth to psychology by applying the paradoxical principle of treating suffering as the foundation for sustainable well-being, as illustrated by Leung’s (2019) tragic optimism of restoring hope through accepting and overcoming traumas and Bowers’ (2019) mature happiness through transcending the dark side of life.

In this way, people search for meaning as a natural part of their life and this search for meaning can lead people to new opportunities and challenges. In contrast, some authors see search for meaning as a symptom of malfunctioning. For example, Baumeister (1991) and Klinger (1998) note that search for meaning is only for people who feel disappointed while trying to meet their needs. Focusing on meaning in life, Frankl (1992) states that meaning alleviates the negative outcomes of painful events encountered by people. Meaning in life is accepted as a positive trait, which is an indicator of well-being (Ryff, 1989). Jones (1995) stresses that search for meaning is one of the most important indicators of mental health. Frankl (1992) argues that the meaning in life can change from time to time, but it never disappears. There are different ways of finding meaning in life. These can be engaging in a task, interacting with people, living goodness, righteousness, beauty, spending time in nature and loving a person. When the explanations made about the meaning in life are taken into consideration, it is seen that the meaning in life does not have a single and universal definition, but it is a concept related to many concepts including especially positive psychology.

Meaning in life is also expressed as an indicator of well-being (Kashdan and Steger, 2007). In the literature, it is stated that meaning in life is related to life satisfaction (Pan et al., 2008); resilience and well-being (Lightsey, 2006); positive and negative well-being (Scannell et al., 2002). Having a sense of meaning in life can play a key role to sustain and maintain psychological health of those who suffer from problems. Meaningful living has become a central topic in existential positive psychology (PP 2.0; Wong, 2011, 2019) which is characterized by a balanced understanding of the good life, incorporating the dynamic interaction between positives and negatives, meaning-centered, and culture.

Well-being enriches and energizes life; it also endows life with a sense of joy and meaning. However, psychology of well-being needs to study both the perils of happiness (Wong, 2007) and the benefits of suffering (Frankl, 1992; Wong, 2009). A complete theory of well-being needs to take into account negative emotions and suffering (in cited Wong, 2011).

Meaning in life and hope contribute positively to an individual’s life satisfaction and increase his well-being in difficult times. Like these variables, fear contributes to the individual’s self-protection by staying alert in difficult times. Although the individual experiences intense fear, positive factors such as meaning in life and hope can increase the person’s self-protection. Therefore, in the regression model created in this study, the fear of COVID-19 and the variables of meaning in life and hope were taken together. The results of the present study are expected to provide guidance for future studies and researchers. The present study aims to determine whether life satisfaction has significant relationships with meaning in life, hope, COVID-19 fear, age, gender and the presence of people infected with COVID-19 around.

## Materials and Methods

### Research Model

The relational survey model was employed in the present study to investigate the relationships between life satisfaction, meaning in life, hope and COVID-19 fear.

### Study Group

The study group of the present study consists of 1,186 adults with the mean age of 41.04. While selecting the sample, the convenience sampling method, one of the non-random sampling methods, was used. The convenience sampling refers to construction of the sample starting with the most convenient respondents until reaching the sample size desired by the researcher (Büyüköztürk et al., 2016). Study group participants are consists of different cities of different regions of Turkey. These cities include Istanbul, Ankara, Konya, Izmir, Denizli, Muğla, Adana, Burdur, Mersin, Antalya, Malatya, Diyarbakır, and Niğde. The demographic features of the sample constructed with this method are given below.

Of the participants in the study group, 57.80% (*n* = 685) are females and 42.20% (*n* = 501) are males. Of the participants, 21.70% (*n* = 257) are in the age group 18–24, 15.80% (*n* = 187) are in the age group 25-30, 17.50% (*n* = 207) are in the age group 31–37, 14.80% (*n* = 175) are in the age group 38-44, 11.50% (*n* = 136) are in the age group 45–50, 14.80% (*n* = 176) are in the age group 51-60 and 4% (*n* = 48) are in the age group 65 and over. While 8.30% (*n* = 98) of the participants have people infected with COVID-19 around, 91.70% (*n* = 1088) do not have cases infected with COVID-19 around. Of the participants in the study group, 0.6% (*n* = 7) primary school degree, 1.00% (*n* = 12) secondary school degree, 7.70% (*n* = 91) high school degree, 71.20% (*n* = 845) university and 19.50% (*n* = 231) have master’s degree.

### Data Collection Tools

In the current study, a personal information form, the Life Satisfaction Scale, the Meaning in Life Scale, the Dispositional Hope Scale and the COVID-19 Fear Scale were used as the data collection tools.

#### Life Satisfaction Scale

The Life Satisfaction Scale was developed by Diener et al. (1985). The scale was adapted to Turkish culture by Dağlı and Baysal (2016). The scale has a total of five items designed in the form of 5-point Likert scale. Higher scores taken from the scale indicate increasing life satisfaction. The scale has single dimension. The scale explains 68.38% of the total variance. Cronbach alpha coefficient of the Scale was 0.88. Sample items of the scale; “My living conditions are perfect,” “I am satisfied with my life” (Dağlı and Baysal, 2016). In present study, the Cronbach alpha internal consistency coefficient of the scale was found to be 0.87.

#### Meaning in Life Scale

The Meaning in Life Scale was developed by Steger et al. (2006). The scale was adapted to Turkish culture by Demirbaş (2010). The scale has a total of 10 items designed in the form of 7-point Likert scale. Higher scores taken from the scale indicate increasing level of meaning in life. The scale explains 68.00% of the total variance. Cronbach alpha coefficient of the Scale was 0.86. The internal consistencies of its sub-dimensions were; 0.87 for the sub-dimension of presence of meaning in life and 0.88 for the sub-dimension of search for meaning in life. Sample items of the scale; “I am aware of the meaning of my life,” “I’m always looking for the purpose of my life” (Demirbaş, 2010). In the present study, the Cronbach alpha internal consistency coefficient was found to be 0.86 for the whole scale, it was found to be 0.86 for the sub-dimension of presence of meaning in life and 0.90 for the sub-dimension of search for meaning in life.

#### Dispositional Hope Scale

The Dispositional Hope Scale was developed by Snyder et al. (1991). The scale was adapted to Turkish culture by Tarhan and Bacanlı (2015). In the scale, there are a total of 12 items designed in the form of 8-point Likert scale. Higher scores taken from the scale indicate increasing level of hope. The scale explains 61.00% of the total variance. Cronbach alpha coefficient of the Scale was 0.86. Test–retest reliability coefficient was found to be 0.86 for the whole scale and it was found to be 0.81 for the sub-dimension of actuating thinking and 0.78 for the sub-dimension of alternative ways thinking. Sample items of the scale; “A problem has many solutions,” “I reach the goals I set for myself” (Tarhan and Bacanlı, 2015). In the current study, the Cronbach alpha internal consistency coefficient was found to be 0.89 for the whole scale, 0.82 for the sub-dimension of actuating thinking and 0.83 for the sub-dimension of alternative ways thinking.

#### COVID-19 Fear Scale

The COVID-19 Fear Scale was developed by Ahorsu et al. (2020). The scale was adapted to Turkish culture by Satıcı et al. (2020). In the scale, there are a total of 7 items designed in the form of 5-point Likert scale. Higher scores taken from the scale indicate increasing level of COVID-19 fear. The scale has single dimension. The total internal consistency coefficient of the COVID-19 Fear Scale was 0.85. Sample items of the scale; “I am very afraid of coronavirus,” “My hands are sweating when I think of the coronavirus” (Satıcı et al., 2020). In the present study, the Cronbach alpha internal consistency coefficient of the scale was found to be 0.87.

### Data Analysis

Multiple linear regression analysis was used to analyze the data. Multiple regression analysis is a type of analysis used to predict the state of the dependent variable on the basis of two or more independent variables (predictor variables) related to the dependent variable. Multiple regression analysis is used for two different research purposes; estimation and explanation. A theory is required to understand the process of criteria for explanation. Estimation is the best guide to develop measurements for variables (Jeon, 2015).

In order to perform statistical analyses on the collected data, first the data (*n* = 1272) were transferred into SPSS 20.0 program package. A total of 50 forms having significant amount of missing data were excluded from the study. Then, accuracy control was conducted in the dataset and all the values were found to be in the possible range (Tabachnick and Fidell, 2007). Moreover, reverse-coded items were corrected and made ready for the missing data examination.

Then the rate of missing data in the dataset was examined and it was found to be less than 5%. Afterward, it was checked whether the missing data pattern exhibited a random distribution and as the result of Little’s MCAR test was found to be insignificant (*p* = 0.322 > 0.05), it was concluded that the missing data exhibited a random distribution (Little, 1988). As the total rate of missing data was found to be less than 5% and the dataset exhibited a random distribution, missing data assignments were made with expectation maximization (EM) (Tabachnick and Fidell, 2007).

In order to determine the outliers in the data set, univariate and multivariate outlier analyses were conducted. First, *z* test was conducted for univariate outlier analysis, as the sampling size is larger than 100, *z* score in the range between −4.0 and +4.0 was taken as the reference value (Mertler and Vannatta, 2005). A total of 22 cases having *z* score in the range between −4.0 and + 4.0 were found to be univariate outliers and thus they were deleted and fourteen other cases were determined through Mahalanobis distance as multivariate outliers and then were deleted (Tabachnick and Fidell, 2007).

In order to test whether each variable satisfies the normality assumption, Kurtosis and skewness coefficients were checked. The Kurtosis and skewness coefficients were found to be within the reference range varying between −1.0 and +1.0. Thus, it can be said that the data distributed normally (Çokluk et al., 2014).

Durbin–Watson coefficient was used to test autocorrelation. Durbin–Watson value was found to be 2.003 and this value is expected to be ranging between 1.5 and 2.5. In order to determine whether there is a multicollinearity problem, simple (paired) correlations between the variables were checked. As a result of the analysis, the paired correlation values between the variables were found to be lower than 0.90 (Çokluk et al., 2014). Moreover, VIF and CI values were also checked to determine whether there is a multicollinearity problem in the dataset; for all the items, VIF values were found to be lower than 10 (Webster, 1992, as cited in Albayrak, 2005) and CI values were found to be lower than 30 (Gujarati, 1995, as cited in Albayrak, 2005). Thus, it can be said that there is no multicollinearity problem between the variables.

As a result of the controls performed, 1,186 of the collected 1,272 forms were found to have met the parametric conditions necessary for regression analysis and thus in all the analyses to be conducted, the set of data obtained from these 1186 forms were used. Finally, in order to find answers to the research questions, Pearson correlation coefficient analysis was used to determine the relationships between the variables and then Multiple Linear Regression Analysis was conducted to determine the extent to which, meaning in life, hope, COVID-19 fear, age, gender, the presence of people infected with COVID-19 around predict life satisfaction in adults. The categorical variables including gender and the presence of people infected with COVID-19 around were converted into dummy variables by assigning codes as 0 and 1 and they were prepared to be suitable for regression analysis. In this regard, the categories of being a male and the presence of people infected with COVID-19 around were coded as 1. Multiple regression analysis is a type of analysis used to predict the state of the dependent variable on the basis of two or more independent variables (predictor variables) related to the dependent variable (Büyüköztürk, 2014). All these statistical analyses were conducted by using SPSS 20.0 program and the significance level was set to be 0.05.

## Results

Multiple linear regression analysis was conducted to determine the extent to which meaning in life, hope, COVID-19 fear, age, gender and the presence of people infected with COVID-19 around predict life satisfaction in adults. Before conducting the regression analysis, in order to determine whether there is a multicollinearity problem, paired correlation coefficients were calculated between the variables, and the results are presented in Table 1.

**TABLE 1:** Results of the Pearson product-moment correlation coefficients.

	*X̄*	*S*	1	2	3	3a	3b	4	5	6	7
1. Life satisfaction	16.083	3.850	1								
2. Meaning in life	48.088	11.224	0.456**	1							
3. Hope total point	50.340	7.583	0.494**	0.412**	1						
3a. Actuating thinking	24.320	4.226	0.555**	0.433**	0.832**	1					
3b. Alternative ways thinking	26.019	3.952	0.355**	0.327**	0.822**	0.719**	1				
4. COVID-19 fear	18.414	5.639	–0.006	−0.132**	−0.086**	–0.041	−0.120**	1			
5. Age	41.042	13.006	0.168**	0.284**	0.185**	0.218**	0.121**	0.032	1		
6. Gender	0.577	0.494	–0.013	–0.055	−0.099**	−0.082**	−0.103**	0.201**	−0.341**	1	
7. The presence of people infected with COVID-19 around	0.082	0.275	0.003	0.016	–0.016	0.000	–0.031	0.068*	–0.043	0.095**	1

**p < 0.01, *p < 0.05.

In Table 1, there is a positive correlation between life satisfaction of adults and meaning in life (*r* = 0.456, *p* < 0.01), hope total point (*r* = 0.494, *p* < 0.01), actuating thinking (*r* = 0.555, *p* < 0.01), alternative ways thinking (*r* = 0.355, *p* < 0.01) and age (*r* = 0.168, *p* < 0.01). Moreover, there is a positive but insignificant correlation between life satisfaction and the presence of people infected with COVID-19 around (*r* = 0.003, *p* > 0.05). Additionally, there is a negative but insignificant correlation between life satisfaction and COVID-19 fear (*r* = −0.006, *p* > 0.05) and gender (*r* = −0.013, *p* > 0.05). It can be understood that these correlations are not at the level high enough (lower than 0.90) to create a multicollinearity problem in the model constructed (Çokluk et al., 2014). Moreover, when the obtained correlation coefficients were examined, it is seen that there is a medium level correlation between life satisfaction in adults and meaning in life, hope, actuating thinking and alternative ways thinking (0.30 < *r* < 0.70) and that there is a low level and insignificant correlation between life satisfaction and COVID-19 fear, gender and the presence of people infected with COVID-19 around. Also there is a low level and significant correlation between life satisfaction and age (0.00 < *r* < 0.30) (Büyüköztürk, 2014).

The results of the multiple linear regression analysis conducted to determine the extent to which meaning in life, hope, COVID-19 fear, age, gender and the presence of people infected with COVID-19 around predict life satisfaction in adults are presented in Table 2.

**TABLE 2:** Results of the multiple linear regression.

	*R*	*R*^2^	Adjusted *R*^2^	*R*^2^_ch_	*F*	df	*B*	β	*t*	*p*
Constant	0.610	0.372	0.368	0.372	99.706**	7/1178	2.143	–	2.833**	0.005
Meaning in life							0.094	0.274	10.279**	0.000
Actuating thinking							0.461	0.506	14.339**	0.000
Alternative ways thinking							0.089	0.091	2.717**	0.007
COVID-19 fear							0.024	0.035	1.428	0.154
Age							0.000	–0.001	–0.040	0.968
Gender							0.219	0.028	1.105	0.270
The presence of people infected with COVID-19 around							–0.132	–0.009	–0.406	0.685

**p < 0.01, *p < 0.05.

As can be seen in Table 2, meaning in life, the sub-dimensions of hope (actuating thinking and alternative ways thinking), COVID-19 fear, age, gender and the presence of people infected with COVID-19 around together explain 37.20% of the life satisfaction in adults. When the results of the *t*-test related to the significance of the regression coefficients are examined, it is seen that meaning in life (*t* = 10.279, *p* < 0.01), actuating thinking (*t* = 14.339, *p* < 0.01) and alternative ways thinking (*t* = 2.717, *p* < 0.01) are significant predictors of life satisfaction in adults as positively. Besides, it is seen COVID-19 fear (*t* = 1.428, *p* > 0.05), age (*t* = −0.040, *p* > 0.05), gender (*t* = 1.105, *p* > 0.05) and the presence of people infected with COVID-19 around (*t* = −0.406, *p* > 0.05) aren’t significant predictors of life satisfaction in adults.

## Discussion

The current study investigated whether there are significant relationships between life satisfaction and hope, meaning in life and COVID-19 fear and the extent to which life satisfaction is predicted by these variables. According to the results of the study, meaning in life, the sub-dimensions of hope (actuating thinking and alternative ways thinking) significantly predict life satisfaction but COVID-19 fear, age, gender and the presence of people infected with COVID-19 around aren’t significantly predict life satisfaction in adults. The danger is very direct, rapid and unpredictable today due to the epidemic. This affects the whole world. The attention, thoughts and feelings of individuals are mostly controlled by direct danger signals seen in mass media in the form of images and facts. Often, these signals quickly arouse fear responses and increase disaster-related thoughts, leading to an increase in the expectation of the worst possible scenarios. The results of the current study show that hope and meaning in life foster life satisfaction even during an epidemic.

In the current study, COVID-19 fear did not significantly predict life satisfaction. Fear is a strong emotion that affects individuals’ physical responses, cognitive skills, and moods (Satıcı et al., 2020). The absence of positivity is different from the presence of negativity and can be equally important (Wood and Johnson, 2016). Inconsistent with the result of this study, previous studies indicated that fear of COVID-19 was significantly and positively associated with depression, stress as well as negatively associated with resilience (Yıldırım and Arslan, 2020); negatively associated with mental well-being (Satıcı et al., 2020); health anxiety, regular media-use, social media use and risks for loved ones (Mertens et al., 2020). Özmen et al. (2021) examine the relationship between the fear of COVID-19, well-being and life satisfaction perceptions of people aged 18 and over living in Turkey. As a result of their study, the participants’ COVID-19 fear levels were found to be moderate. Also they found a negative and low level of relationship was found between COVID-19 fear and life satisfaction.

Fear is generally a primitive feeling and arises in the face of a real or perceived threat. This feeling is for the present, as it involves producing a response to something that is believed to be threatening (Dozois and Rnic, 2019). The data of this study were not collected at the initial stage of the COVID-19 pandemic, but during the period when the spread was increasing. In this case, the fear may have turned into other negative emotions. Life satisfaction is a general phenomenon and may not be affected very quickly by sudden changes. Since fear is a temporary emotion, its contribution to life satisfaction is lower than other positive variables. In this case, the negative contribution of COVID-19 fear to life satisfaction has decreased with the positive effect of meaning in life and hope variables. Also the low number of infected individuals around people at the time the data were collected in this study may also be the reason why fear of COVID-19 does not predict life satisfaction.

In our study gender, age and the presence of an infected person around did not significantly predict satisfaction with life. There are different research results on gender and life satisfaction during the COVID-19 pandemic. According to the findings, coronavirus pandemic causes more psychological effects in females (Wang D. et al., 2020) and females have less life satisfaction than males (Raza et al., 2020). Additionally, our finding supports the finding of Karataş and Tagay (2020) that gender was not significant predictors of the level of resilience in adults. consistent with the result of this study Helliwell et al. (2020) indicated that, the equality of male and female life satisfaction responses held across the life satisfaction response scale, so that well-being inequality rose equally for both men and women. We detected age was not significantly predict satisfaction with life. The WHO reported that individuals 50 and above are at higher risk for coronavirus-related death than those in any other age groups. Specifically, coronavirus related death is more common among individuals 60 years of age and above. In current research the study group average age 41.04 so this may be the reason why age does not significantly predict life satisfaction. Also the results of the study show that presence of an infected person around did not significantly predict life satisfaction. Our finding supports the finding of Karataş and Tagay (2020) that presence of an infected person around was not significant predictors of the level of resilience in adults.

The results of the study show that, individuals with higher levels of life satisfaction have higher levels of hope. While a high level of hope is an indicator of good health and full functioning, its low level is interpreted as an indicator of personal sadness and distress (Martin, 2007). People’s evaluations about whether they can achieve their goals affect their feelings and believing that efforts will lead to success increases the level of hope. Experiences of pain are a condition that harms people and challenges resources. It is stated that strong people who can cope with the elements threatening the social order are more hopeful. This is an important psychological strength (Nesse, 1999). Through this psychological strength, individuals cope with stressful situations more easily and their satisfaction with life is positively affected (Snyder, 2002).

The Dispositional Hope Scale used in the current study has two dimensions: actuating thinking and alternative ways thinking. Accordingly, it is seen that it is important to set a goal, to work to achieve this goal and to determine alternative ways to reach the goal when confronted with obstacles to be hopeful (Tarhan and Bacanlı, 2015). As is known, life satisfaction is one of the important concepts of positive psychology and is related to hope. Valle et al. (2006) argue that higher levels of hope increase life satisfaction and Cole (2008) states that life satisfaction is related to how individuals are close to their goals and that being closer to the accomplishment of goals increases life satisfaction while distancing from these goals because of any obstacle decreases life satisfaction.

In the current study meaning in life positively and significantly predicts life satisfaction. Despite the differences in the definitions of and ways to meaning in life, theorists regard meaning in itself as vital. Meaningful life is directly related to authentic life (Kenyon, 2000) and psychological strengths (Ryff and Singer, 1998). Frankl (1963) argues that people are characterized by a desire for meaning, an innate urge to find meaning in their lives, and failure to achieve meaning leads to psychological distress. Also, according to Frankl, the meaning alleviates the stress caused by the painful events that people face in their lives (Ryff, 1989). Thus, people may experience difficulties due to the uncertainty and fear involved in a pandemic. But, having meaning in such periods increases life satisfaction.

### Implications and Limitations

The COVID-19 pandemic has shown people how quickly the world has changed during the pandemic. People can also quickly adapt to this change process. The contribution of such studies will be high in terms of revealing what we need to deal with the difficulties of a pandemic more easily. According to the results of the current study, individuals with more hopeful and meaningful lives have higher life satisfaction. Also similar studies can be carried out about idea could be to turn everything around and, for example, look at how hope, meaning, life satisfaction and perhaps knowing someone infected prognoses’ COVID-19 fear. The current study was conducted with adults. Similar studies can be carried out with children and adolescents. In addition, it is thought that it is important to increase the training activities aimed at increasing life satisfaction in order to protect public mental health.

Findings of this study should also be considered in the light of a few methodological limitations. First, self–reported measure was used to collect data from adults (the average age 41.04); therefore, future research should examine the variables using different data collection approaches (e.g., qualitative). Second, participants included adults (the average age 41.04) so considering this limitation, further studies could be conducted using diverse and large samples (e.g., children, adolescents, university students). The majority of the participants of this study is people with higher education and lives in big cities. Therefore, the results of the study are limited to this group. It can be suggested that new studies will be conducted with people with different socio-economic levels and education. The cross-sectional approach is considered as another limitation of the study, and longitudinal research is warranted to examine the causal relationship between the variables of the study. In the current participants’ group the level of the COVID-19 fear were rather low and only 8% of participants have infected people around them. So the results of this research can be generalized to similar groups.

Finally, data were collected using self-reported measures which are subjected to participants’ biases.

## Data Availability Statement

The raw data supporting the conclusions of this article will be made available by the authors, without undue reservation.

## Ethics Statement

The studies involving human participants were reviewed and approved by the Burdur Mehmet Akif Ersoy University Ethics Committee. The patients/participants provided their written informed consent to participate in this study.

## Author Contributions

ZK contributed to the research design, data collection, data analysis, interpretation, and intellectual content. KU contributed to the data collection, data analysis, and intellectual content. ÖT contributed to the research design, interpretation, and intellectual content. All authors contributed to the article and approved the submitted version.

## Conflict of Interest

The authors declare that the research was conducted in the absence of any commercial or financial relationships that could be construed as a potential conflict of interest.

## Footnotes

1 https://covid19.who.int/
